# Feasibility of ossicular chain reconstruction with resin cement^☆^

**DOI:** 10.1016/j.bjorl.2016.02.014

**Published:** 2016-04-29

**Authors:** Fernando de Andrade Quintanilha Ribeiro, Yumi Tamaoki, Gabriel Wynne Cabral

**Affiliations:** Irmandade da Santa Casa de São Paulo, Departamento de Otorrinolaringologia, São Paulo, SP, Brazil

**Keywords:** Ossicular prosthesis, Cyanoacrylates, Resin cements, Prótese ossicular, Cianoacrilatos, Cimentos resinosos

## Abstract

**Introduction:**

Disjunction of ossicular chain is a common finding in middle ear chronic disease. In addition to ossicular interposition, various materials have been used for reconstruction, such as ceramic prostheses, polyethylene, and titanium.

**Objective:**

Because of the high cost of the available options, the authors propose to reconstruct the ossicular chain with resin cement, a material typically used in dental reconstruction and fixation.

**Methods:**

Two anatomical parts of the temporal bones were used, creating a disjunction of the ossicular chain between the incus and staples and then reconstructing with resin cement. These reconstructions were repeated four times by three different surgeons to ensure the feasibility of the method.

**Results:**

A total of 12 reconstructions were carried out, four per surgeon. After applying the cement, it could be verified by touch that the space was filled properly by the used material. Proper articulation with motion transfer to the entire ossicular chain was also observed.

**Conclusion:**

Resin cement is a suitable material in the reconstruction of ossicular chain injury, and it is inexpensive and technically simple.

## Resumo

**Introdução:**

É frequente a disjunção da cadeia ossicular nas doenças crônicas da orelha média. Além de interposições ossiculares, vários materiais já foram utilizados com a finalidade de reconstruí-la, como próteses de cerâmica, polietileno e titânio.

**Objetivo:**

Devido ao alto custo das opções existentes, propomos reconstruir a cadeia com cimento resinoso, material usado normalmente na reconstrução e fixação dentária.

**Método:**

Serão utilizadas duas peças anatômicas de ossos temporais, onde será criada uma disjunção da cadeia entre a bigorna e o estribo que, a seguir, será reconstruída com o cimento resinoso. Estas reconstruções serão repetidas quatro vezes por três cirurgiões diferentes, para certificação da viabilidade do método.

**Resultados:**

Foram feitas um total de 12 reconstruções, quatro por cada cirurgião. Após aplicação do cimento, conseguimos, ao toque, perceber que o espaço foi preenchido adequadamente pelo material empregado. Notamos ainda adequada articulação com transferência de movimento para toda a cadeia ossicular.

**Conclusão:**

O cimento resinoso é um material viável na reconstrução de lesões da cadeia ossicular, e proporciona um método tecnicamente simples e de baixo custo.

## Introduction

Despite isolated reports dating from the 19th and early 20th centuries, the development of glue and self-adhesive substances for use in health care had its real momentum after 1940, with the use of plasma enriched with heterologous and homologous fibrinogen in the manufacture of biological adhesives.^1^ The first description of the use of synthetic adhesives in medicine dates back to 1958, when epoxy (or epoxyline) was used to join bone tissue in experimental fractures.^2^ But it was from the 1960s onwards, with the advent of cyanoacrylates, that research using synthetic adhesives in medicine had its big boost.^3^

Originally discovered in 1949 by Harry Coover, cyanoacrylates were commercially released only in 1958, and in 1959 its applicability was reported for closing tissue injuries.^3^, ^4^ They are commonly used in medicine and have proven their applicability in clinical and experimental studies. In otorhinolaryngology, specifically in the area of ear surgery, experiments with the use of self-adhesive substances began around 1960. The main areas of application focused on the use of adhesives for soft tissue union, such as skin, fascia, and tympanic membrane, ossicular chain reconstruction, and adherence between prostheses and middle ear structures.^5^ Another derivative of cyanoacrylate widely investigated for use in otologic surgery was 2-butyl-cyanoacrylate or Histoacryl. In the early 1970s, experiments with dogs used Histoacryl to join the temporal fascia to the tympanic membrane, create an interposition between the bone/cartilage and stapes footplate, and create an interposition between hammer and stirrup. These experiments indicated that Histoacryl, when used in small amounts, was not harmful to the inner and middle ear structures and degraded in a short period of time, creating a stable union between tissues.^5^ Other derivatives of cyanoacrylates were also used to repair facial nerve in dogs^6^ and in revision surgeries for fixing a stapedectomy prosthesis to the remainder of the anvil, with good functional results.^7^

As for the materials available for ossicular chain reconstruction, autologous structures such as tympanic ossicles, mastoid cortical bone, and tragal cartilage are still the most used options, despite the emergence of various metal prostheses, such as gold, steel wire, platinum, and titanium; plastics, such as silicone, polyethylene, polytetrafluoroethylene; and ceramics, such as ceramic oxide, carbon, calcium phosphate ceramic, hydroxyapatite, and glazed ceramic.^8^, ^9^, ^10^ All these synthetic materials have in common the disadvantage of being expensive, making their use impractical in many centers. More recently, the introduction of ceramics and cements commonly used in restoration and dental fillings has been reported in otology with a goal to perform ossicular chain reconstruction more quickly and easily. Among these, approximately ten years ago the use of ceramic glass ionomer was relatively well studied, but the results are still very variable and sometimes inconsistent.^11^

Resin cement was first developed in dentistry as an alternative to conventional cementation. The introduction of this new material offered dentists a new tool for cementing that was easier to use than previous materials, and had great adhesiveness and esthetics.^12^ Currently, various resin cements have been introduced in the market and are classified into two categories. Conventional resin cements, which do not have an inherent adhesion to tooth structure and require the use of an adhesive system; and the self-adhesive resin cements, which do not require a prior adhesive treatment of dental substrate.^12^, ^13^ The RelyX Unicem – 3M ESPE^®^ was the first self-adhesive resin cement, commercially introduced in 2002. However, other brands are already available, differing in the form of presentation, color, and chemical composition. These products have two pastes requiring manipulation that may be manual by trituration in capsules or by a self-mixing dispenser. There is little information on the adhesion mechanism of adhesive cements. The material's basic composition is the monomeric system Bis-GMA (Bisphenol-A glycidyl methacrylate^®^) in combination with low viscosity monomers, in addition to inorganic fillers (glass with metallic charge, SiO_2_) treated with silane.^14^ They have high adhesiveness, biocompatibility, are ease to handle, and have a simple-to-use protocol.^14^ Although they are already widely used in dentistry with excellent results, having been indicated for bonding various substrates such as enamel, dentin, amalgam, metal, and porcelain, there are few studies regarding its clinical performance in this area, and no study in the medical field.

In otology, the erosion of the long arm of the incus is a frequent finding. In a quest for a more anatomical reconstruction, the authors assessed the feasibility of ossicular chain reconstruction with resin cement, an accessible and inexpensive material.

## Methods

Two surgical specimens of human temporal bones will be used, officially provided by the Department of Morphology of the institution, which will be handled in the surgical training session in temporal bones of the Department of Otolaryngology. After fixation in a dissection basin, the tympanomeatal flap will be folded down and the incudo-stapedial joint exposed. After the disarticulation of the incudostapedial joint, the distal portion of long process of the incus will be cut with iris scissors, so that the distance between the remaining long process and the stapes superstructure is approximately 3 mm. This is the distance typically found in clinical cases of disarticulation by necrosis of the distal portion of the long arm of the incus.

The resin cements that will be used are the Relix^®^ and Bifix^®^ preparations, usually found in dental supply stores. Bifix^®^ has a mixer coupled to the tube, but it uses more material. It has the advantage of a fine nozzle that can deposit the mixture directly into the space to be filled and costs approximately U$45 (USD). Relix^®^ without the mixer is cheaper and can be used in more occasions; its cost is approximately U$42.50 (USD). The chain integrity must be redone using the abovementioned materials, which will be interposed with the use of a spatula.

The procedure will be performed by three surgeons with different levels of experience: a professor, a post-graduate, and a resident. Each will perform the procedure four times, and the difficulties regarding cement handling, intervention time, and interposition functioning will be analyzed. As the cement interposition can be removed through a careful mechanical procedure, it can be remade several times in the available parts.

## Results

The mixing time of the used materials was 40 s. The maximum time available to fill the bone interruption with the materials and its appropriate modeling before hardening was 1 min. Thus, a creamy white bridge was formed between the ossicles, mimicking the normal bone. By moving both the malleus and the incus, it was observed that the movement was properly transferred to the stapes. Table 1 shows the procedures performed.

**Table 1:** Procedures performed.

Participants	1st attempt	2nd attempt	3rd attempt	4th attempt
Professor	Hardening of the material during the process	Inadequate transmission of material to stirrup	Bridge functionally adequate	Bridge functionally adequate
Post-graduate	Inadequate transmission of material to stirrup	Bridge functionally adequate	Bridge functionally adequate	Bridge functionally adequate
Resident	Hardening of the material during the process	Hardening of the material during the process	Bridge functionally adequate	Bridge functionally adequate

## Discussion

**Figure 1:**
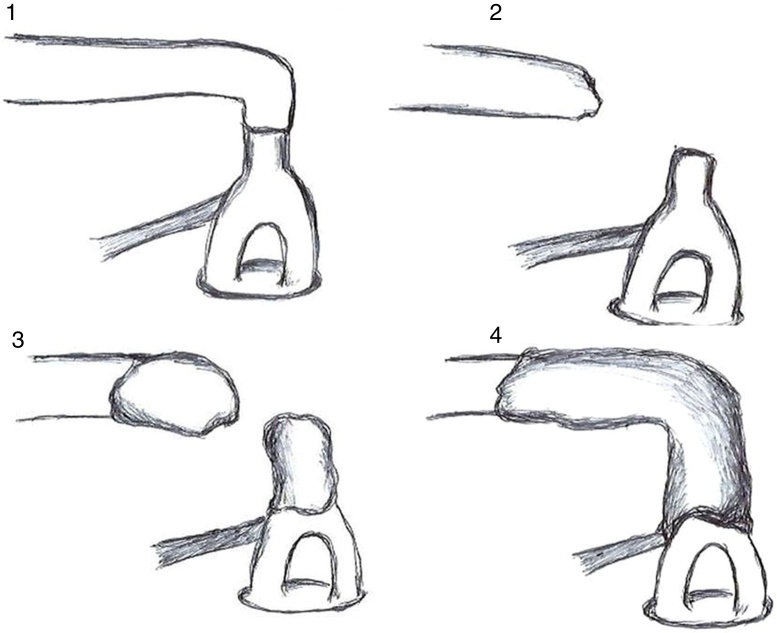
Schematic drawing of the four attempts at ossicular chain reconstruction with resin cement.

As shown in Table 1, the procedure was initially performed by the professor, who had difficulty in managing the time between the mixing of materials and their proper placement in filling the disjunction. This was due to an attempt to deposit the material only with the use of a spatula, in which it had great adherence; this resulted in rapid hardening. In the second attempt, with the concomitant use of a pointer, the material was removed from the spatula in small quantities and taken to the area to be filled. The material was then placed interchangeably in the stapes and incus to complete the filling, as shown in Fig. 1.

Subsequently, it was noted that the bridge did not conduct the movement from the incus to the stapes well, because the material did not adhere to the stapes well. In the third attempt, a larger amount of material was brought to the proximity of the stapes tendon, extending through the long process of the incus and partially surrounding it; this led to an adequate transmission of the movement. It was also noted that the material adhered well to the ossicles, without reaching the footplate region, which could lead to its fixation. The same occurred at the fourth attempt.

The then-established procedure was performed by the otolaryngologist, who had difficulty in properly positioning the material on the stirrup on the first attempt, but did it correctly in subsequent attempts. In the first two attempts, the resident faced early hardening of the material, but properly handled it in the two subsequent attempts.

Thus, three surgeons with different experiences in otologic surgery managed, with little initial difficulty and rapid learning, to promote the restructuring of the ossicular chain injured by the erosion of the long process of the incus using resin cement. The same material can possibly be used in other procedures of ossicular chain reconstruction. However, additional studies should be conducted before its application in human otologic surgery. In this line, there are ongoing experiments with guinea pigs to evaluate resin cement toxicity and hearing restoration after its use through BERA.

## Conclusion

Resin cement is a viable material for ossicular injury reconstruction.

The reconstruction method is achieved with little training by surgeons.

The process is financially advantageous.

## Funding

This study was approved by the Ethics Committee of the institution, registered under the No. 969.234 12/03/2015.

## Conflicts of interest

The authors declare no conflicts of interest.
